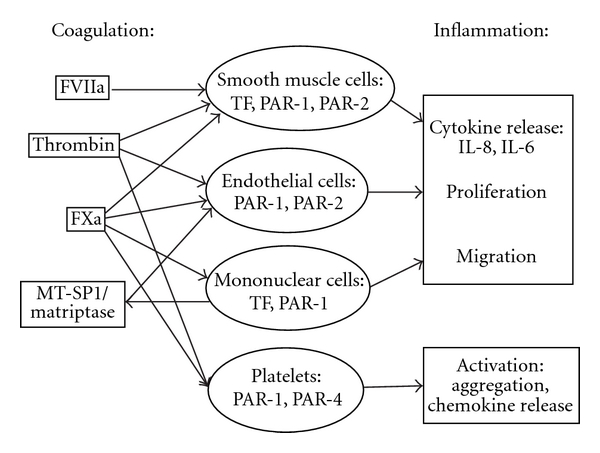# The Interface between Inflammation and Coagulation in Cardiovascular Disease

**DOI:** 10.1155/2012/860301

**Published:** 2012-02-19

**Authors:** Gabriele Demetz, Ilka Ott

**Affiliations:** Deutsches Herzzentrum Technische Universität München, Lazarettstraße 36, 80636 München, Germany

## Abstract

The intimate connection between coagulation and inflammation in the pathogenesis of vascular disease has moved more and more into focus of clinical research. This paper focuses on the essential components of this interplay in the settings of cardiovascular disease and acute coronary syndrome. Tissue factor, the main initiator of the extrinsic coagulation pathway, plays a central role via causing a proinflammatory response through activation of coagulation factors and thereby initiating coagulation and downstream cellular signalling pathways. Regarding activated clotting factors II, X, and VII, protease-activated receptors provide the molecular link between coagulation and inflammation. Hereby, PAR-1 displays deleterious as well as beneficial properties. Unravelling these interrelations may help developing new strategies to ameliorate the detrimental reciprocal aggravation of inflammation and coagulation.

## 1. Introduction

Systemic and local proinflammatory changes are in focus when investigating the pathophysiology of arteriosclerosis and acute coronary syndromes. In acute myocardial infarction (AMI), proinflammatory markers such as C-reactive protein (CRP), interleukins, or monocyte-chemoattractant protein (MCP)-1 are elevated [1–3] and their increase is of prognostic relevance for future cardiovascular events [4–6] and mortality [7–9]. Moreover, in healthy persons elevated proinflammatory markers are associated with an increase in cardiovascular risk [10–12]. Patients with increased circulating proinflammatory markers in AMI present with decreased myocardial salvage after coronary reperfusion therapy [13]. Similarly, in experimental studies, high levels of CRP deteriorate infarct size [14].

Sources of inflammatory response are vascular cells such as activated endothelial cells, which release proinflammatory cytokines such as interleukin (IL)-8 [15]. IL-8 is a CXC cytokine that acts as a chemoattractant and agonist for neutrophils, lymphocytes, and monocytes and is found in macrophage-rich atherosclerotic plaques [16]. Under flow conditions, IL-8 facilitates the arrest of monocytes on endothelium [16], which is necessary for migration into the intima in evolution of arteriosclerosis. Reperfusion injury after AMI as well as systemic inflammatory response syndrome can be associated to increased levels of IL-8 [17]. In experimental setting, murine IL-8 receptor knock-out mice display smaller arteriosclerotic lesions with less macrophages [18]. Apart from their contribution to arteriosclerosis, CXC cytokines are also produced by malignant cells and can promote tumor progression of a large variety of malignancies [19].

Besides IL-8, many other cytokines such as IL-6 take part in inflammatory responses by inducing B-cell differentiation, T-cell activation, and synthesis of acute phase proteins [20], but also contributing to proliferation of vascular smooth muscle cells (SMCs) [21]. Moreover TH1 activation was observed in acute coronary syndromes [22].

The pathogenesis of proinflammatory changes in acute coronary syndromes as well as the interplay between coagulation and inflammation is poorly understood and is subject to intense research. The mechanisms by which the coagulation system is altered by inflammatory interactions comprise enhanced synthesis and activation of coagulant proteins, decreased synthesis of anticoagulants, and suppression of fibrinolysis [23]. However, not only inflammation activates coagulation but coagulation in turn perpetuates inflammatory response [24]. Accordingly, increased levels of prothrombin fragment F1+2, fibrinopeptide A and D-Dimer, reflecting activation of the coagulation cascade, are also associated with an unfavorable outcome in acute coronary syndromes [25–27].

In this paper we focus on possible mechanisms of the interplay between coagulation and inflammation in acute coronary syndromes.

## 2. Tissue Factor

A strict separation between the intrinsic and extrinsic coagulation cascade surely fails to reflect physiologic conditions. Nevertheless, in the setting of arteriosclerosis and acute coronary syndromes the extrinsic pathway of coagulation is of particular significance.

Tissue Factor (TF) is the most important initiator of the extrinsic coagulation cascade. TF, a 47 kDa transmembrane glycoprotein and member of the class II cytokine receptor family, is the cofactor for the activated plasma clotting factor VII (FVIIa). The TF-FVIIa complex catalyzes the activation of factor X and IX, which leads to the generation of thrombin and thus finally of a fibrin clot. Under physiologic conditions TF is abundantly expressed only in the adventitia and is induced by several inflammatory mediators such as IL-6, IL-8, and MCP-1 [28, 29]. After vascular injury, TF is rapidly augmented in SMC of the media and accumulates in the SMC of the developing neointima [30]. Consequently, TF is highly expressed within atherosclerotic lesions and displays high procoagulant activity suggesting a role in determining plaque thrombogenicity [30]. In atherosclerotic carotid lesions disruption of plaques exposes TF-positive cells within the plaque to plasma clotting factors and initiates local thrombosis with subsequent occlusion of the vessel [31].

Furthermore, increased TF expression can be noticed on circulating monocytes and microparticles in acute coronary syndromes and may, thereby, contribute to activation of coagulation [32–34]. A soluble form of TF within the circulating blood may also support coronary thrombosis [35]. It has been shown that cytokines can induce expression of soluble TF [36], which on the other hand has been shown to accumulate in developing thrombi [37]. However, the clinical significance and individual contributions of microparticle-derived and soluble TF remain a matter of debate. Several studies have demonstrated increased levels of circulating TF in patients with unstable Angina pectoris (uAP) and acute myocardial infarction (AMI) [32, 38–45]. Therefore, it has long been speculated that, in cases with no plaque rupture or only fractional superficial erosion, thrombus formation may mainly depend on circulating levels of TF. Consistent with this idea, several studies suggest that the levels of circulating TF and other haemostatic biomarkers may correlate to adverse cardiovascular events and mortality in patients with acute coronary syndrome [46–48].

Stimulation of the TF-thrombin pathway does not only occur at the site of the plaque but also within the ischemic myocardium where activated coagulation factors may enhance inflammatory responses and increase infarct size [49]. TF contributes to inflammation, cell migration, and remodelling after vascular injury [50]. Furthermore, TF expression has been reported in a number of cancers, such as glioma, pancreatic cancer, non-small-cell lung cancer, colorectal cancer, ovarian cancer, prostate cancer, hepatocellular cancer, and breast cancer [51]. TF expression in tumors not only correlates with the incidence of thrombosis [52] but also promotes metastasis [53], tumor progression, and tumor angiogenesis [54].

TF-mediated intracellular signal transduction has not been completely elucidated so far. On one hand TF allows docking and activation of FVII and, therefore, promotes the generation of downstream coagulation factors and activation of protease-activated receptors (PARs) which themselves possibly induce intracellular signal transduction. On the other hand there is evidence for direct signalling through the cytoplasmic domain of TF following TF-FVIIa complex formation [55, 56].

## 3. Tissue Factor Pathway Inhibitor

The endogenous Kunitz-type inhibitor Tissue Factor Pathway Inhibitor-1 (TFPI) inhibits initiation of TF-induced blood coagulation and is mainly expressed on vascular endothelial cells. TFPI binds and inactivates FXa. The TFPI-FXa complex then binds and inactivates FVIIa. Increased levels of the TFPI-FXa complex may reflect both increased FXa generation and increased TFPI concentrations [57]. In addition to the full length TFPI most of the plasma TFPI circulates in truncated forms that are bound to plasma lipoproteins. These truncated forms lack their C-terminal domains and exhibit reduced affinity for vascular wall proteolysis. Additionally, it has been shown that endogenous proteases [58] and elastase released by neutrophils degrade TFPI, resulting in enhanced local coagulation that contributes to prevent pathogen dissemination during infection [59]. Conversely, infusion of a mutant TFPI protein resistant to proteolysis by elastase strongly impaired host defence against systemic infection.

## 4. Protease-Activated Receptors

Important players in the interaction between coagulation and inflammation are protease-activated receptors (PARs). PARs are G-protein coupled receptors that mediate various cellular reactions as cytokine release, expression of adhesion molecules, cell migration, or proliferation. Unlike other receptors, PARs are not activated by a soluble, external ligand. Proteases, such as activated coagulation factors, detach a defined part of the NH2-terminal chain of the receptor, thereby inducing a conformational change of the receptor. This change causes a self-activation by a “tethered ligand.” This activating sequence comprises only few amino acids. PARs can also be activated by synthetic peptides consisting of the sequence of amino acids representing the tethered ligand.

In contrast to other receptors, the activation of PARs by enzymatic cleavage is irreversible. After proteolytic activation the receptor must be internalized, degraded, and resynthesized. PARs are mainly expressed in vascular cells, but also in many different other cell types such as gastrointestinal and bronchial epithelial cells. Four different PARs are known: PAR-1, -3, and -4 show responsible for thrombin signaling whereas PAR-2 is activated by trypsin-like serine proteases, FVIIa, and matriptase but not by thrombin. PAR-1 and PAR-2 are expressed on smooth muscle cells and endothelial cells, whereas mainly PAR-1 is expressed on monocytes.

PAR-1 agonists or thrombin induce IL-8 and IL-6 in SMC, EC, and mononuclear cells (MNCs) [60] therefore emphasizing the role of PAR-1 in inflammatory processes in vascular cells and confirming data about PAR-1-mediated cytokine release in EC and monocytes [61]. In smooth muscle cells PAR-1 and PAR-2 agonists induce cytokine release to a similar extent which underlines the relevance of both PARs [60]. In addition to coagulation factors other serine proteases, for example, matriptase secreted by monocytes stimulate proinflammatory cytokine release in endothelial cells via PAR-2 activation [62]. Increased PAR-2 expression in atherosclerotic lesions suggests a role for this proinflammatory pathway (Figure 1) [63].

In addition, PARs can also be cleaved downstream of the tethered ligand, resulting in receptor inactivation by preventing further proteolytic activation [64]. PAR-1 signalling not only induces inflammatory responses but also causes antiapoptotic and vasculoprotective reactions [65]. Since the anticoagulant protease-activated protein C can activate PAR1 when in complex with the endothelial cell protein C receptor (EPCR), which may account for much of the protective effects conferred by activated protein C (APC) in severe sepsis [66]. Different contributions of these two pathways may prevail. First, APC acts via PAR-1 when attached to EPCR [65], resulting in cellular responses distinct from thrombin signalling [64] by a mechanism dependent in trans-activation of the sphingosine 1 phosphate receptor 1. In mouse models with strongly reduced EPCR expression or PAR-1 deficiency, the loss of EPCR/APC signalling via PAR-1 resulted in increased endotoxemia-induced lethality [67]. Concordantly, APC mutants have been shown to contribute to protective effects during sepsis by pathways independent from anticoagulant properties [67, 68]. The second pathway described is independent from EPCR. In this case, the availability of the integrin CD11b/CD18 has been shown to be crucial for PAR-1 mediated APC signaling on macrophages, thereby exhibiting anti-inflammatory effects and reducing endotoxin-induced lethality [69]. Thus the strength of PAR1 and PAR2 activation by thrombin, factor Xa, and activated protein C can either promote or protect against changes in vascular permeability depending on the status of the endothelium.

Platelet activation with subsequent thrombus generation plays a major role in the development of acute coronary syndromes. At low concentrations thrombin activates PAR-1 on platelets through a hirudin-like site and at high concentrations additional PAR-4. This induces shape change, P-selectin, and CD40L mobilization to the platelet membrane and promotes the release of platelet agonists ADP, thromboxane A2, chemokines, and growth factors [70] and, thereby, enhances proinflammatory changes. Thus, inhibition of PARs by thrombin of FXa inhibitors may prove beneficial in reducing not only thrombotic but also proinflammatory responses.

## 5. FXa

Binding of the serine protease FVII to TF results in generation of the coagulation protease FXa (FXa) and subsequently thrombin both known to induce cell signaling. FXa shows dose-dependent induction of intracellular calcium transients in endothelial cells that is active-site-dependent, and independent of thrombin [71]. Potential pathophysiological responses to FXa include stimulation of proliferation, production of proinflammatory cytokines, and prothrombotic TF [72].

Elevated TFPI-FXa and prothrombin fragments F1+2 plasma levels indicate activation of the coagulation cascade in acute coronary syndromes. Under physiological conditions an inverse relationship between TFPI-FXa and F1+2 suggests that TFPI-FXa regulates prothrombinase activity in vivo [73]. Under conditions associated with activation of the coagulation cascade, however, increased TFPI-Xa plasma levels occur [57, 74]. Activation of coagulation as measured by TFPI-FXa but not by F1+2 is associated with plasma concentrations of the proinflammatory cytokine IL-8 in acute coronary syndromes [60]. Furthermore, subsequent elevated IL-6 levels in the course of acute coronary syndromes are associated with initial TFPI-FXa concentrations [60]. These results argue for a proinflammatory role of FXa in acute coronary syndromes that is independent of thrombin. Although thrombin provokes similar proinflammatory effects as FXa in vitro the effects of thrombin may be diminished after heparin treatment in vivo. Several trials of unfractionated heparin (UFH) [75], direct thrombin inhibitors [76], and enoxaparin [77] have thus far failed to demonstrate mortality reductions in acute coronary syndromes. Yet, the OASIS-6 trial suggests a reduction in reinfarction and mortality without excess bleeding in patients not undergoing PCI [78]. Therapeutic inhibition of the proinflammatory effects of Factor Xa may, therefore, prove additional benefits as compared to thrombin inhibition in the clinical course in acute coronary syndromes. This is currently investigated in the ATLAS-ACS 2 TIMI 51 trial that is testing the hypothesis that anticoagulation with the oral factor Xa inhibitor rivaroxaban reduces cardiovascular death, MI, and stroke among patients with ACS treated with guideline-based therapies for ACS [79].

In vitro experiments revealed that FXa stimulates IL-8 and MCP-1 transcription in endothelial cells and mononuclear leukocytes [60]. Genetic studies and receptor desensitization experiments indicate that signaling by FXa is mediated by PAR-1 and PAR-2 [80, 81].

According to the expression of PAR-1 and PAR-2, PAR-1 and PAR-2 agonists induce IL-8 and MCP-1 release in endothelial cells, whereas, only PAR-1 agonists stimulated cytokine release in mononuclear cells [60].

## 6. FVIIa

Several studies suggest a signaling mechanism of the TF-FVIIa complex via PAR-2 [82]. In this model, TF-bound FVIIa proteolytically activates PAR-2 and, to a lesser extent, PAR-1, and thereby evokes intracellular signaling cascades [83].

In contrast to FXa, FVIIa does not elicit a proinflammatory response in endothelial cells or mononuclear cells [82]. The TF-FVIIa-PAR-2 signalling was only observed in SMC [82] since they express both TF and PAR-2 and both of them seem to be a prerequisite for FVIIa action. EC expressing PAR-2 but lacking TF will not permit FVIIa docking, whereas MNC displaying TF but only low PAR-2 expression, probably allow FVIIa binding but do not express sufficient PAR-2 molecules being subsequently activated. However, PAR-2 and TF are induced by cytokine stimulation [28, 84]. Thus, FVIIa may still have an important impact in atherosclerotic vessels [31] and acute coronary syndromes [29].

In recent studies [85], it has been demonstrated that FVII is synthesized by different cancer cells (liver, ovary, prostate, lung, gastric, thyroid, and breast). Considering that these tumor cells also synthesize TF it is conceivable that supraphysiologic concentrations of FVIIa after binding of FVII to TF occur. On tumor cells, the TF-FVIIa binary complex mediates activation of PAR-2 [86]. Therefore TF-FVIIa-PAR-2 interaction with subsequent cytokine release may be relevant within a tumor environment. TF/FVIIa/PAR2 signalling has been shown to promote proliferation and metastasis of tumor cells [87, 88]. Consistently, TF/FVIIa-specific upregulation of IL-8 expression in breast cancer cells has been shown to be mediated by PAR-2 and to increase cell migration [83]. Whether TF-FVIIa-PAR-2 interaction may also contribute to local thrombus formation and progression of atherosclerotic disease remains to be elucidated.

## 7. Conclusion

The interplay between coagulation and inflammation is a matter of intense research. Proinflammatory changes in acute coronary syndromes may substantially influence prognosis [6]. Experimental evidence suggests that this interplay may contribute to the development of vascular remodeling or support plaque disruption of the artery. However transfer of these data to the clinical settings remains controversial since additional optimized medical and interventional treatments interfere. Therefore, understanding the causes of inflammation facilitate the development of new therapeutic strategies. To analyse whether these new therapies translate to improved clinical outcome needs to be studied in appropriate clinical trials.

While inhibiting proinflammatory cytokines such as Tumor-Necrosis Factor-*α* (TNF) has been shown to effectively improve survival in several animal models of sepsis [89–91], anti-TNF therapy in septic humans failed to ameliorate or even worsened clinical outcome [92–95]. In chronic inflammatory diseases however, anti-inflammatory treatment has become clinical routine. For example, inhibition of IL-6 by the first anti-IL-6 antibody, tocilizumab, has been shown to completely block TF-dependent thrombin generation in experimental endotoxemia [23, 96], and tocilizumab will be of special future interest as it has been approved for rheumatoid arthritis.

Inhibiting coagulation could depict a more promising mechanism in fighting overwhelming inflammatory response. Experimental studies that have shown that anticoagulant treatment not only diminishes activation of coagulation but also inhibits inflammation, underline the cross-talk between activation of coagulation and cytokine release in vivo [49, 97, 98]. In acute inflammatory disorders such as severe sepsis, administration of recombinant APC significantly improves survival and long-term outcome. In future, APC mutants that lack anticoagulant properties but still enable sphingosine 1 phosphate receptor 1 dependent activation of PAR-1 will be of special clinical interest as they have been shown to reduce sepsis-induced in mice but do not predispose to bleeding complications.

So far, anti-inflammatory treatments displayed no explicit benefit in patients with acute coronary syndromes [99]. However, there is evidence that FXa inhibitors prove to be superior to thrombin inhibitors [100]. Particularly, treatment with low molecular weight heparins, that include additional anti-FXa activity as compared to unfractionated heparin, has been shown to decrease inflammatory changes in vitro and in vivo [101, 102].

Yet the question remains if and what anticoagulant therapies will prove beneficial to alter systemic inflammatory responses.

## Figures and Tables

**Figure 1:**